# Supplementary material for: In Situ Generation of Green Hybrid Nanofibrillar Polymer-Polymer Composites—A Novel Approach to the Triple Shape Memory Polymer Formation
Source: Polymers (Basel). 2021 Jun 8;13(12):1900. doi: 10.3390/polym13121900 (PMC8226873; doi:10.3390/polym13121900)
Supplement: Supplementary file 1 [file polymers-13-01900-s001.zip › polymers-1238370-supplementary.pdf]

Supplementary Materials

Table S1. The method Log for dual-SME and triple-SME.

Method Log for dual-SME	Method Log for triple-SME
1: Force 0.003 N<br>2: Ramp 10.00°C/min to 65.00°C<br>3: Isothermal for 5.00 min<br>4: Data storage: On<br>5: Ramp strain 2.000 %/min to 10.000 %<br>6: Ramp 10.00°C/min to 20.00°C<br>7: Isothermal for 10.00 min<br>8: Force 0.003 N<br>9: Isothermal for 5.00 min<br>10: Ramp 10.00°C/min to 65.00°C<br>11: Isothermal for 45.00 min<br>12: Data storage: Off<br>13: End of method	1: Force 0.003 N<br>2: Ramp 10.00°C/min to 120.00°C<br>3: Isothermal for 2.00 min<br>4: Data storage: On<br>5: Ramp strain 2.000 %/min to 10.000 %<br>6: Ramp 10.00°C/min to 65.00°C<br>7: Isothermal for 2.00 min<br>8: Ramp strain 2.000 %/min to 25.000 %<br>9: Ramp 10.00°C/min to 20.00°C<br>10: Isothermal for 10.00 min<br>11: Force 0.003 N<br>12: Isothermal for 5.00 min<br>13: Ramp 10.00°C/min to 65.00°C<br>14: Isothermal for 30.00 min<br>15: Ramp 10.00°C/min to 120.00°C<br>16: Isothermal for 45.00 min<br>17: Data storage: Off<br>18: End of method

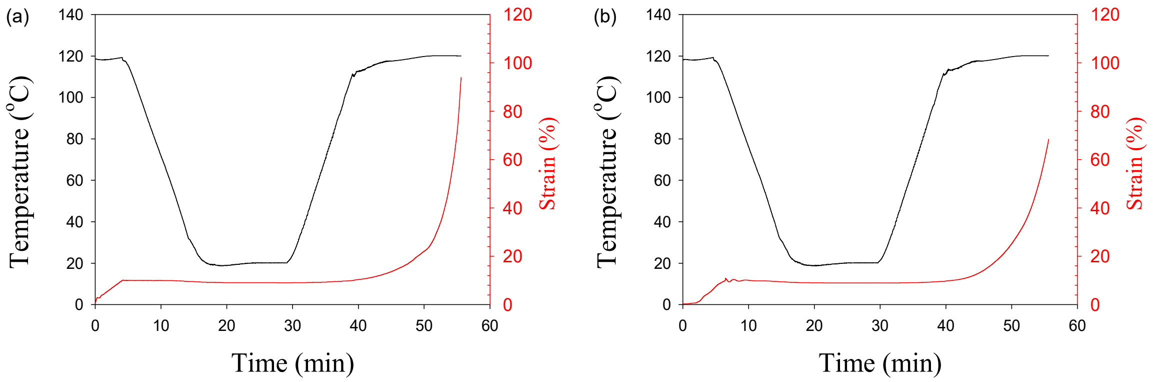

Figure S1:Temperature and strain of PLA/PBAT/CNFs blends (a) and in-situ generated composites (b) during one-way shape memory cycle. T_d=120 °C. CNFs - 3 wt.%.
